# Efficacies of Colistin–Carbapenem versus Colistin–Tigecycline in Critically Ill Patients with CR-GNB-Associated Pneumonia: A Multicenter Observational Study

**DOI:** 10.3390/antibiotics10091081

**Published:** 2021-09-07

**Authors:** Sheng-Huei Wang, Kuang-Yao Yang, Chau-Chyun Sheu, Wei-Cheng Chen, Ming-Cheng Chan, Jia-Yih Feng, Chia-Min Chen, Biing-Ru Wu, Zhe-Rong Zheng, Yu-Ching Chou, Chung-Kan Peng, on behalf of the T-CARE (Taiwan Critical Care Infection) Group

**Affiliations:** 1 Division of Pulmonary and Critical Care Medicine, Department of Internal Medicine, Tri-Service General Hospital, National Defense Medical Center, Taipei 114, Taiwan; slidersinker@gmail.com; 2 Graduate Institute of Medical Sciences, National Defense Medical Center, Taipei 114, Taiwan; 3 Department of Chest Medicine, Taipei Veterans General Hospital, Taipei 112, Taiwan; kyyang@vghtpe.gov.tw (K.-Y.Y.); peterofeng@gmail.com (J.-Y.F.); 4 Institute of Emergency and Critical Care Medicine, School of Medicine, National Yang Ming Chiao Tung University, Taipei 112, Taiwan; 5 Cancer Progression Research Center, National Yang Ming Chiao Tung University, Taipei 112, Taiwan; 6 Division of Pulmonary and Critical Care Medicine, Department of Internal Medicine, Kaohsiung Medical University Hospital, Kaohsiung Medical University, Kaohsiung 807, Taiwan; sheucc@gmail.com (C.-C.S.); kmuronald@gmail.com (C.-M.C.); 7 Department of Internal Medicine, School of Medicine, College of Medicine, Kaohsiung Medical University, Kaohsiung 807, Taiwan; 8 Graduate Institute of Biomedical Sciences, China Medical University, Taichung 404, Taiwan; pion501@gmail.com; 9 Division of Pulmonary and Critical Care Medicine, Department of Internal Medicine, China Medical University Hospital, Taichung 404, Taiwan; aloumayxxx@gmail.com; 10 Department of Education, China Medical University Hospital, Taichung 404, Taiwan; 11 Division of Critical Care and Respiratory Therapy, Department of Internal Medicine, Taichung Veterans General Hospital, Taichung 407, Taiwan; mingcheng.chan@gmail.com; 12 National Chung Hsing University, Taichung 402, Taiwan; 13 School of Medicine, National Yang Ming Chiao Tung University, Taipei 112, Taiwan; 14 Ph.D. Program in Translational Medicine, National Chung Hsing University, Taichung 402, Taiwan; 15 Rong Hsing Research Center for Translational Medicine, National Chung Hsing University, Taichung 402, Taiwan; 16 Division of Pulmonary Medicine, Department of Internal Medicine, Chung Shan Medical University Hospital, Taichung 402, Taiwan; rockfenix0329@gmail.com; 17 Division of Chest Medicine, Department of Internal Medicine, Taichung Veterans General Hospital, Taichung 407, Taiwan; 18 School of Public Health, National Defense Medical Center, Taipei 114, Taiwan; trishow@mail.ndmctsgh.edu.tw

**Keywords:** carbapenem resistant, colistin, tigecycline, pneumonia, nephrotoxicity

## Abstract

**Background:** Evaluating the options for antibiotic treatment for carbapenem-resistant Gram-negative bacteria (CR-GNB)-associated pneumonia remains crucial. We compared the therapeutic efficacy and nephrotoxicity of two combination therapies, namely, colistin + carbapenem (CC) versus colistin + tigecycline (CT), for treating CR-GNB-related nosocomial pneumonia in critically ill patients. **Methods:** In this multicenter, retrospective, and cohort study, we recruited patients admitted to intensive care units and diagnosed with CR-GNB-associated nosocomial pneumonia. We divided the enrolled patients into CC (*n* = 62) and CT (*n* = 59) groups. After propensity score matching (*n* = 39), we compared the therapeutic efficacy by mortality, favorable outcome, and microbiological eradication and compared nephrotoxicity by acute kidney injury between groups. **Results:** There was no significant difference between the CC and CT groups regarding demographic characteristics and disease severities as assessed using the Acute Physiology and Chronic Health Evaluation (APACHE) II score, Sequential Organ Failure Assessment (SOFA) score, and other organ dysfunction variables. Therapeutic efficacy was non-significantly different between groups in all-cause mortality, favorable outcomes, and microbiological eradication at days 7, 14, and 28; as was the Kaplan-Meier analysis of 28-day survival. For nephrotoxicity, both groups had similar risks of developing acute kidney injury, evaluated using the Kidney Disease Improving Global Outcomes criteria (*p* = 1.000). **Conclusions:** Combination therapy with CC or CT had similar therapeutic efficacy and risk of developing acute kidney injury for treating CR-GNB-associated nosocomial pneumonia in critically ill patients.

## 1. Introduction

Carbapenem-resistant Gram-negative bacteria (CR-GNB) including carbapenem-resistant *Acinetobacter baumannii* (CRAB), carbapenem-resistant *Pseudomonas aeruginosa* (CRPA), and those under carbapenem-resistant Enterobacteriaceae (CRE) have been placed by the World Health Organization (WHO) in the global priority list of antibiotic-resistant bacteria in 2016 [1]. CR-GNB (especially CRAB and CRPA)-associated infections occur predominantly in the intensive care unit (ICU), and the problem of CR-GNB persists in developed and developing countries [2,3,4,5]. CRAB predominantly infects debilitated patients in the ICU and is one of the major pathogens causing hospital-acquired pneumonia (HAP) and ventilator-associated pneumonia (VAP), thereby resulting in high morbidity and mortality worldwide [6,7,8]. 

Colistin, tigecycline, carbapenem, and sulbactam are common antibiotics used for the treatment of CR-GNB-associated infections, and other agents including amikacin, minocycline, rifampicin, fosfomycin, and trimethoprim-sulfamethoxazole are prescribed occasionally [9,10]. Colistin is the main backbone of combination therapy for CR-GNB infection, and many studies observed a synergistic effect developed when colistin combined with carbapenem or tigecycline [11,12,13]. By definition, CR-GNB is resistant to carbapenem therapy. However, the combination of colistin and carbapenem develops the synergistic effect, wherein colistin changes the permeability of the bacterial outer membrane, allows a high amount of carbapenem to penetrate into the bacteria, and then becomes effective against CR-GNB [11,14]. As for tigecycline, the synergistic effect with colistin may result from disruption of the bacterial outer membrane and unstable status of the cytoplasmic membrane due to colistin that facilitated uptake and accumulation of tigecycline in the cytoplasm for the binding of the ribosomal complex [15]. Furthermore, although most CR-GNBs are susceptible to tigecycline, tigecycline is usually prescribed as combination regimens when treating CR-GNB-associated pneumonia for its insufficient steady maximum concentration in the epithelial lining fluid of the lung [16,17].

Although many randomized controlled trials (RCTs), cohort studies, and meta-analyses have tried to determine the best regimen for CR-GNB treatment, there is no conclusive guideline to follow in clinical practice [18,19,20,21]. The therapy for CR-GNB infection has been proposed according to the infectious site and disease severity, and a combination of two in vitro active antibiotics was suggested for the treatment of CR-GNB-associated HAP and VAP [5]. However, the direct comparison of two combination therapies, namely, colistin + carbapenem (CC) versus colistin + tigecycline (CT), has not been investigated previously. In this study, we conducted a multicenter, retrospective, cohort study to compare the therapeutic efficacy of these two combination regimens (CC vs. CT) in ICU patients infected with CR-GNB-associated HAP/VAP.

## 2. Methods

### 2.1. Study Population and Setting

**Figure 1:**
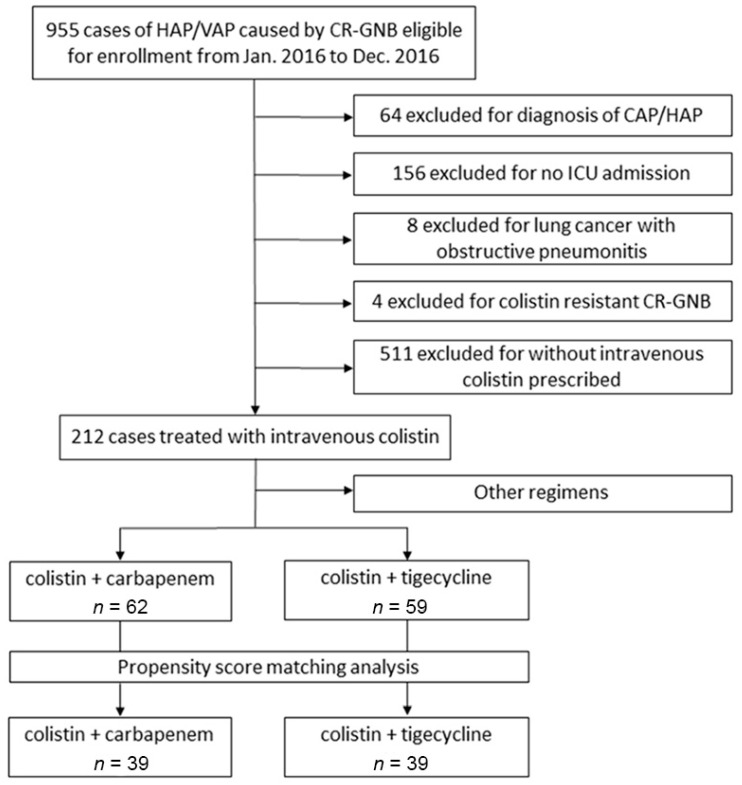
Flowchart showing study patient inclusion and exclusion.

This multicenter, retrospective, cohort study was conducted in five medical centers in Taiwan from January 2016 to December 2016. Relevant studies on this topic have been published or are in process [22]. A flowchart for study patient inclusion and exclusion is shown in Figure 1. The inclusion criteria included the following: (1) ICU-admitted patients who were diagnosed with HAP or VAP. (2) Respiratory specimens with CR-GNB in cultures that were resistant to at least one kind of carbapenem. Exclusion criteria included age <20 years; diagnosis of community-acquired pneumonia (CAP) or health-care-associated pneumonia (HCAP); lung cancer with obstructive pneumonitis; colistin-resistant CR-GNB; and no prescription of intravenous colistin within 7 days of the index date of pneumonia. Finally, patients treated with a combination therapy of colistin + carbapenem or colistin + tigecycline were recruited for analysis. 

### 2.2. Data Collection of Baseline Characteristics

Data on demographic characteristics and baseline variables were retrieved from patients’ medical records. Disease severity was evaluated using the Acute Physiology and Chronic Health Evaluation (APACHE) II score on the day of ICU admission and the Sequential Organ Failure Assessment (SOFA) score on the day of ICU admission and pneumonia index date. Data on other variables associated with organ dysfunction were also collected on the pneumonia index date, including septic shock, mechanical ventilator use, PaO_2_/FiO_2_ (P/F) ratio, and renal replacement therapy (hemodialysis + continuous venovenous hemofiltration).

### 2.3. Diagnosis of Pneumonia and Microbiological Tests

Pneumonia was diagnosed when there was presence of progressive disease or new infiltration on chest radiography accompanied by at least two of the following clinical findings: hyperthermia (>38 °C) or hypothermia (<36 °C), cough, purulent sputum production, leukocytosis (plasma white cell count >10,000 per mm^3^), leukopenia (plasma white cell count <4000 per mm^3^), or band cell percentage of >10%. Eligible specimens were collected from the sputum, tracheal aspirates, or bronchoalveolar lavage fluid with a CR-GNB concentration of >10^4^ colony-forming units per milliliter. The pneumonia index date (pneumonia onset day) was defined as the date of specimen collection. The determination of susceptibility to carbapenems was confirmed according to the Clinical and Laboratory Standards Institute recommendations.

### 2.4. Therapeutic Regimens

All patients in this study were treated with intravenous colistimethate sodium, a prodrug, which was hydrolyzed to its active from (colistin) in plasma. Intravenous antibiotics that were prescribed within 7 days of the pneumonia index date with a duration of ≥2 days were recorded. The colistin + carbapenem (CC) group and colistin + tigecycline (CT) group were defined by the concurrent prescription of colistin and carbapenem or colistin and tigecycline, respectively, with a duration of ≥2 days. The selection and dosage of antibiotics were determined by the specialized clinicians according to the clinical condition of patients. We compared therapeutic efficacy and nephrotoxicity between the CC group and the CT group, and the concurrently administered antibiotics for CR-GNB treatment, including inhaled colistin, intravenous sulbactam, and amikacin, were also analyzed. Carbapenems prescribed in the current study included meropenem, imipenem, and doripenem. 

### 2.5. Outcomes and Nephrotoxicity Evaluations

The primary outcomes evaluated in this study were mortality rate, clinical response, and microbiological response at days 7, 14, and 28. The clinical response to treatment was classified as cure (resolution of symptoms and free from antibiotics), improvement (partial resolution of symptoms but not free from antibiotics), or failure (persistent symptoms or death). Both cure and improvement were defined as clinically favorable outcomes. Microbiological responses were classified as eradication (no growth of causative pathogens in at least two consecutive respiratory specimens), persistence (persistent growth of causative pathogens in respiratory specimens), recurrence (re-isolation of causative pathogens within 14 days of eradication), and undetermined (follow-up specimen unavailable or only one specimen with no growth). The microbiological eradication rate was defined as the ratio of the number of cases of eradiation to the sum of the number of cases of eradiation, persistence, and recurrence (not including undetermined).

Secondary outcomes included the length of hospital stay, length of ICU stay, 28-day ventilator weaning rate, and nephrotoxicity. The assessment of hospital and ICU stays did not include patients who died during hospitalization. We evaluated nephrotoxicity based on the development of acute kidney injury (AKI), which was defined according to the Kidney Disease Improving Global Outcomes (KDIGO) criteria (creatinine increase ≥0.3 mg/dL within 2 days or ≥50% from baseline within 7 days). AKI analysis did not include patients who were receiving renal replacement therapy at baseline or had insufficient creatinine data to enable AKI assessment.

### 2.6. Propensity-Score Matching Analysis

For minimizing the differences in demographic characteristics and disease severity between the CC and CT groups, a propensity-score-matching (PS-matching) analysis was performed with 1:1 matching and a 0.2 caliper width to investigate the primary and secondary outcomes. The PSs were calculated by the logistic regression of variables including age, sex, pneumonia types, heart failures, lung diseases, and diabetes.

### 2.7. Statistical Analysis

Data on continuous variables are expressed as the means ± standard deviations, and those on categorical variables are expressed as percentages. Continuous variables were compared with the Mann–Whitney U test, while categorical variables were compared with the chi-square test or Fisher’s exact test. Cox and logistic regression analyses were performed to identify the independent factors associated with mortality on day 28 and clinically favorable outcomes and microbiological eradication on day 14. Kaplan–Meier analysis and log-rank tests were used to compare 28-day survival between the CC and CT groups. Statistical analyses were performed using SPSS, version 18.0 (SPSS Inc., Chicago, IL, USA). A *p* value of ≤0.5 was considered statistically significant. This study was approved by the Institutional Review Boards of all the participating hospitals (registration numbers: 2018-03-001CC, 1-107-05-054, CE18100A, CMUH107-REC3-052, and KMUHIRB-E(I)-20180141).

## 3. Results

### 3.1. Demographic Characteristics and Disease Severities before and after PS Matching

Table 1 shows the demographic characteristics of the CC (*n* = 62) and CT (*n* = 59) groups before PS matching. CRAB was the predominant pathogen in both groups. The CC group (82.3%) had a significantly higher proportion of patients who were diagnosed with VAP than the CT group (57.6%, *p* = 0.006). There was no significant difference regarding the comorbidities between the CC and CT groups except heart failure (6.5% vs. 22.0%, *p* = 0.028), lung disease (11.3% vs. 30.5%, *p* = 0.017), and diabetes (22.6% vs. 47.5%, *p* = 0.007), which were significantly more common in the CT group than in the CC group. Patients were recorded with the comorbidity of lung disease if they were diagnosed with chronic obstructive pulmonary disease, asthma, bronchiectasis, active tuberculosis, or interstitial lung disease. In addition, there was no significant difference between groups regarding the co-administered antibiotics, including inhaled colistin, sulbactam, and amikacin. Furthermore, we evaluated disease severity by parameters including APACHE II score, SOFA score, septic shock, invasive ventilator, P/F ratio, and dialysis, but there was no significant difference between groups. For laboratory data analysis, the CT group had a significantly higher leukocyte count than the CC group (15,487.63 vs. 12,784.52, *p* = 0.027). After PS matching (Table 2), there were no significant differences in demographic characteristics and disease severity between CC (*n* = 39) and CT groups (*n* = 39). 

### 3.2. Therapeutic Efficacy

**Figure 2:**
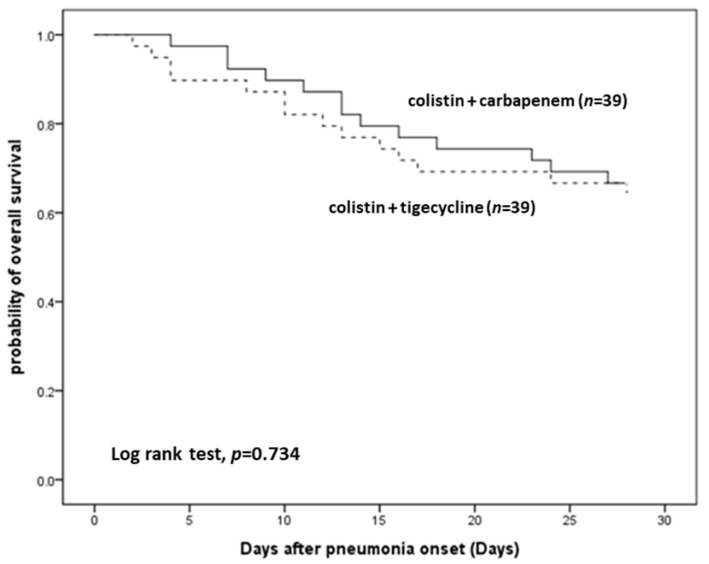
Kaplan–Meier survival curves for patients treated with colistin + carbapenem or colistin + tigecycline after propensity score matching.

Table 3 shows the comparison of therapeutic efficacy between the CC and CT groups after PS matching. In primary outcomes, there were no significant differences in all-cause mortality, favorable clinical outcomes, and microbiological eradication on days 7, 14, and 28. There were still no significant difference in both groups for secondary outcomes including length of hospital stay, length of ICU stay, and 28-day ventilator weaning condition. In Table 4, we observed that neither the CC nor CT group was an independent factor for 28-day all-cause mortality, favorable clinical outcomes on day 14, or microbiological eradication on day 14 by multivariate analysis. In Figure 2, Kaplan–Meier analysis of 28-day survival did not show significant differences between the CC and CT groups.

### 3.3. Nephrotoxicity

In Table 3, we assessed nephrotoxicity by AKI development after the initiation of combination therapy. A total of 53.6% and 50.0% patients in CC and CT group, respectively, developed AKI, but we did not observe significant differences between these two groups. 

## 4. Discussion

This multicenter and retrospective study observed similar therapeutic efficacy by comparing all-cause mortality, favorable clinical outcomes, and microbiological eradication between CC and CT combination treatment for CR-GNB nosocomial pneumonia. The results were confirmed in PS-matching analysis and multivariate analysis. We also assessed nephrotoxicity based on AKI, and the risk of developing AKI is similar in both groups. 

The in-hospital mortality rate of CC therapy in our study is 51.3%, which is similar to those reported in Shi’s (55.4%, colistin + carbapenem) [14] and Katip’s (54.96%, colistin + meropenem) [23] studies. The 28-day mortality rate of CC therapy in our study is 33.3%, and it is lower than the rate reported in Paul’s RCT (45%) [24]. The high mortality rate reported in Paul’s study might be attributed to 47% of patients receiving colistin + meropenem therapy for bacteremia. As for microbiological eradiation, CC therapy at day 14 was also similar to Shi’s study (46.4% vs. 41.0%) [14]. Moreover, 53.6% of our patients developed AKI with CC therapy, which was higher than the percentage reported in Katip’s (49.62%) study [23]. The disparity between studies could result from different criteria to define nephrotoxicity (KDIGO vs. RIFLE) and diverse disease severity at baseline. 

Scarce clinical studies compared the combination therapy between CC and CT for the treatment of nosocomial pneumonia [25,26]. Chaari reported that there was no significant difference in the mortality at day 28 between the colistin–tigecycline and colistin–imipenem combination treatments for *Acinetobacter baumannii*-associated ventilator-acquired pneumonia (hazard ratio = 0.76, 95% confidence interval 0.44–1.33; *p* = 0.34), which was consistent with the findings of our study, although the small case number (total *n* = 79) may lack the power to identify possible disparity [25]. Khawcharoenporn performed a comparison of colistin-based therapy for extensively drug-resistant *Acinetobacter baumannii* pneumonia and reported the survival rate at 28 days and the microbiological cure at the end of therapy were not significantly different between the CC and CT combination, which is similar to the observations of our study [26]. Furthermore, consistent with the findings of our study, the risk of developing AKI was similar between CC and CT combination in Khawcharoenporn’s study, and the KDIGO criteria were applied to define AKI in both studies [26].

As for microbiology eradication, a recent systematic review and meta-analysis conducted by Mei observed the microbiological eradication rate was lower in the tigecycline groups compared to other therapeutic agents [27]. Although it was not statistically significant, the microbiology eradication rate in our study was lower in the CT group than the CC group at day 7 and 14. However, even with lower microbiology eradication rate in the CT regimen, the addition of colistin to tigecycline maintained a similar mortality rate as the CC group, so did the Khawcharoenporn’s study [26].

This study has some merits. First, the multicenter study could consider different settings of clinical practice in different hospitals and decrease the possibility of selection bias. Second, PS-matching analysis was adopted to minimize the baseline differences between CC and CT groups, which contributed to the strengths of the primary and secondary outcomes. Third, limited studies have evaluated the therapeutic efficacy and nephrotoxicity of the colistin + tigecycline combination therapy, and our study provides additional information and a thorough view for clinical clinicians. However, some limitations exist in this study. First, the number of cases of this study may not be sufficient, which may have resulted in limited statistical power to differentiate the disparity in the CC and CT groups. However, from another point of view, we observed that low NNT (numbers needed to treat) could not discern the difference between these two regimens for the small sample size of our study. Furthermore, we applied PS-matching analysis to minimize this flaw. Second, most of the enrolled cases had CRAB-associated nosocomial pneumonia, so others should be cautious to apply our finding to non-CRAB CR-GNB-associated nosocomial pneumonia. Third, all patients included in the present study were treated in the ICU; therefore, the findings cannot be extrapolated to other clinical settings. Fourth, we only recorded the all-cause mortality by the initial study design, so we could not further analyze the cause of each death case.

In conclusion, the addition of carbapenem or tigecycline to intravenous colistin resulted in similar therapeutic efficacy (evaluated by all-cause mortality, favorable clinical outcomes, and microbiological eradication) and nephrotoxicity (assessed by AKI in critically ill patients). RCTs are warranted to scrutinize this finding and evaluate the best therapeutic regimen for CR-GNB-associated pneumonia. 

## Figures and Tables

**Table 1:** Demographic characteristics and disease severity of ICU patients treated with colistin + carbapenem or colistin + tigecycline.

	Colistin + Carbapenem (*n* = 62)	Colistin + Tigecycline (*n* = 59)	*p* Value
Age, M (SD)	66.63 (18.07)	69.24 (12.62)	0.357
Sex, *n* (%)			0.868
Female	24 (38.7)	21 (35.6)	
Male	38 (61.3)	38 (64.4)	
Height, M (SD)	163.44 (9.98)	161.68 (8.74)	0.330
Weight, M (SD)	60.44 (13.11)	63.20 (16.21)	0.336
BMI, M (SD)	22.48 (3.63)	24.07 (5.46)	0.075
Smoking	23 (37.7)	23 (39.7)	0.976
Alcohol consumption	13 (21.7)	7 (11.9)	0.236
Pathogen, *n* (%)			0.102
CR-Pseudo	3 (4.8)	3 (5.1)	
CRAB	57 (91.9)	48 (81.4)	
CRKP	2 (3.2)	8 (13.6)	
Pneumonia types, *n* (%)			0.006
HAP	11 (17.7)	25 (42.4)	
VAP	51 (82.3)	34 (57.6)	
ICU types, *n* (%)			0.649
Medical ICU	44 (71.0)	45 (76.3)	
Surgical ICU	18 (29.0)	14 (23.7)	
Comorbidities			
Lung cancer, *n* (%)	6 (9.7)	2 (3.4)	0.274
Malignancy	10 (16.1)	6 (10.2)	0.485
Liver disease	6 (9.7)	8 (13.6)	0.702
Heart failure	4 (6.5)	13 (22.0)	0.028
Hypertension	29 (46.8)	32 (54.2)	0.523
Stroke	9 (14.5)	7 (11.9)	0.871
Degenerative brain disease	7 (11.3)	4 (6.8)	0.585
Renal insufficiency	11 (17.7)	16 (27.1)	0.308
Lung disease	7 (11.3)	18 (30.5)	0.017
Diabetes	14 (22.6)	28 (47.5)	0.007
Autoimmune disease	3 (4.8)	5 (8.5)	0.484
Coadministered antibiotics			
Inhaled colistin, *n* (%)	26 (41.9)	21 (35.6)	0.597
Sulbactam	8 (12.9)	8 (13.6)	1.000
Amikacin	1 (1.6)	1 (1.7)	1.000
Disease severity			
APACHE II score, M (SD)	22.74 (8.93)	21.70 (8.13)	0.396
SOFA score (ICU admission date), M (SD)	8.18 (3.76)	8.71 (3.82)	0.543
SOFA score (pneumonia index date), M (SD)	8.34 (3.45)	8.00 (3.71)	0.508
Septic shock	10 (16.1)	13 (22.0)	0.551
Invasive ventilator	52 (83.9)	56 (94.9)	0.095
PF ratio, M (SD)	268.62 (130.44)	267.46 (113.51)	0.907
Dialysis (HD + CVVH) Lab data analysis	14 (22.6)	7 (11.9)	0.188
Leukocyte, M (SD)	12,784.52 (8319.27)	15,487.63 (8722.18)	0.027
Neutrophil, M (SD)	10,670.78 (6904.97)	12,784.51 (7195.77)	0.031
C-reactive protein, M (SD)	15.08 (29.77)	11.90 (9.19)	0.974
Albumin, M (SD)	2.61 (0.61)	2.51 (0.55)	0.188
Creatinine, M (SD)	2.10 (1.79)	2.06 (1.81)	0.989

M (SD): Mean (standard deviation).

**Table 2:** Demographic characteristics and disease severity of ICU patients treated with colistin + carbapenem or colistin + tigecycline after propensity score matching.

	Colistin + Carbapenem (*n* = 39)	Colistin + Tigecycline (*n* = 39)	*p* Value
Age, M (SD)	71.72 (17.94)	68.00 (14.05)	0.304
Sex, *n* (%)			0.245
Female	18 (46.2)	12 (30.8)	
Male	21 (53.8)	27 (69.2)	
Height, M (SD)	162.03 (9.63)	162.94 (8.98)	0.687
Weight, M (SD)	58.25 (12.09)	64.44 (15.64)	0.075
BMI, M (SD)	22.10 (3.64)	24.10 (4.82)	0.061
Smoking	13 (33.3)	16 (42.1)	0.576
Alcohol consumption	8 (20.5)	6 (15.4)	0.768
Pathogen, *n* (%)			0.588
CR-Pseudo	1 (2.6)	3 (7.7)	
CRAB	36 (92.3)	33 (84.6)	
CRKP	2 (5.1)	3 (7.7)	
Pneumonia types, *n* (%)			1.000
HAP	11 (28.2)	12 (30.8)	
VAP	28 (71.8)	27 (69.2)	
ICU types, *n* (%)			1.000
Medical ICU	29 (74.4)	28 (71.8)	
Surgical ICU	10 (25.6)	11 (28.2)	
Comorbidities			
Lung cancer, *n* (%)	2 (5.1)	2 (5.1)	1.000
Malignancy	5 (12.8)	3 (7.7)	0.711
Liver disease	4 (10.3)	5 (12.8)	1.000
Heart failure	4 (10.3)	6 (15.4)	0.735
Hypertension	21 (53.8)	19 (48.7)	0.821
Stroke	8 (20.5)	6 (15.4)	0.768
Degenerative brain disease	6 (15.4)	4 (10.3)	0.735
Renal insufficiency	11 (28.2)	11 (28.2)	1.000
Lung disease	7 (17.9)	8 (20.5)	1.000
Diabetes	14 (35.9)	10 (25.6)	0.462
Autoimmune disease	3 (7.7)	4 (10.3)	1.000
Coadministered antibiotics			
Inhaled colistin, *n* (%)	16 (41.0)	13 (33.3)	0.639
Sulbactam	5 (12.8)	7 (17.9)	0.754
Amikacin	1 (2.60)	1 (2.6)	1.000
Disease severity			
APACHE II score, M (SD)	22.72 (9.58)	20.19 (7.69)	0.216
SOFA score (ICU admission date), M (SD)	8.54 (3.70)	8.21 (4.40)	0.718
SOFA score (pneumonia index date), M (SD)	8.38 (3.45)	8.10 (4.04)	0.741
Septic shock	5 (12.8)	7 (17.9)	0.754
Invasive ventilator	31 (79.5)	36 (92.3)	0.193
PF ratio, M (SD)	245.12 (123.90)	266.68 (109.21)	0.439
Dialysis (HD + CVVH) Lab data analysis	8 (20.5)	5 (12.8)	0.543
Leukocyte, M (SD)	12,022.82 (7664.57)	14,000.77 (7233.81)	0.245
Neutrophil, M (SD)	10,028.23 (6385.35)	11,547.83 (6035.16)	0.262
C-reactive protein, M (SD)	16.97 (36.13)	11.51 (9.35)	0.411
Albumin, M (SD)	2.73 (0.60)	2.51 (0.57)	0.107
Creatinine, M (SD)	2.04 (1.90)	1.90 (1.86)	0.740

M (SD): Mean (standard deviation).

**Table 3:** Therapeutic efficacy and acute kidney injury in the colistin + carbapenem and colistin + tigecycline groups after propensity score matching.

	Colistin + Carbapenem (*n* = 39)	Colistin + Tigecycline (*n* = 39)	*p* Value
Mortality (since pneumonia onset)			
Day 7, *n* (%)	3 (7.7)	4 (10.3)	1.000
Day 14, *n* (%)	8 (20.5)	9 (23.1)	1.000
Day 28, *n* (%)	13 (33.3)	14 (35.9)	1.000
In-hospital mortality, *n* (%)	20 (51.3)	16 (41.0)	0.496
Favorable clinical outcomes			
Day 7	20 (51.3)	19 (48.7)	1.000
Day 14	21 (53.8)	20 (51.3)	1.000
Day 28	18 (46.2)	22 (56.4)	0.497
Microbiological eradication			
Day 7	5 (25.0)	3 (15.8)	0.695
Day 14	13 (46.4)	7 (33.3)	0.529
Day 28	18 (60.0)	12 (57.1)	1.000
Length of hospital stay (days), M (R)	62 (14–284) (*n* = 19)	55 (27–134) (*n* = 23)	0.390 ^a^
Length of ICU stay (days), M (R)	26 (9–95) (*n* = 19)	21 (7–101) (*n* = 23)	0.487 ^a^
28-day ventilator weaning	10 (55.6) (*n* = 18)	10 (43.5) (*n* = 23)	0.651
Acute kidney injury	15 (53.6)	16 (50.0)	0.986

M (R): Median (range); ^a^ Mann–Whitney U test; MV: Mechanical ventilation. The assessment of hospital and ICU stays did not include patients who died during hospitalization. Definition of acute kidney injury: creatinine increase ≥0.3 mg/dL within 2 days or ≥50% from baseline within 7 days according to the KDIGO criteria. The comparison of AKI did not include the patients who were receiving renal replacement therapy at baseline and those who lacked adequate creatinine data for the assessment of AKI.

**Table 4:** Multivariate analysis of clinical factors associated with treatment outcomes after propensity score matching.

	28-Day All-Cause Mortality ^a^		Favorable Clinical Outcomes on Day 14 ^b^		Microbiological Eradication Day 14 ^b^	
	aHR (95% CI)	*p* Value	aOR (95% CI)	*p* Value	aOR (95% CI)	*p* Value
Colistin + tigecycline (vs. colistin + carbapenem)	1.15 (0.53–2.49)	0.722	0.79 (0.31–2.01)	0.626	0.60 (0.18–1.94)	0.388
Age	0.98 (0.96–1.00)	0.110	1.01 (0.98–1.04)	0.751	1.01 (0.97–1.04)	0.654
Male	0.69 (0.32–1.52)	0.359	2.44 (0.94–6.35)	0.069	0.64 (0.20–2.05)	0.449

^a^ Adjusted hazard ratio (aHR) and 95% confidence interval (CI) were derived from Cox regression analysis. ^b^ Adjusted odds ratios (aORs) and 95% CIs were derived from logistic regression analysis.

## Data Availability

The data presented in this study are available on request from the corresponding author. The data are not publicly available due to privacy.
